# Introduction to the Special Issue: Exploration of the Activity of Asteroid (101955) Bennu

**DOI:** 10.1029/2020JE006549

**Published:** 2020-09-09

**Authors:** C. W. Hergenrother, C. D. Adam, S. R. Chesley, D. S. Lauretta

**Affiliations:** ^1^ Lunar and Planetary Laboratory University of Arizona Tucson AZ USA; ^2^ KinetX Aerospace Simi Valley CA USA; ^3^ Jet Propulsion Laboratory California Institute of Technology Pasadena CA USA

## Abstract

Near‐Earth asteroid (101955) Bennu is an active asteroid experiencing mass loss. The activity manifests itself in the form of ejection events emitting up to hundreds of millimeter‐ to centimeter‐scale particles. The Origins, Spectral Interpretation, Resource Identification, and Security‐Regolith Explorer spacecraft monitored particle activity for a 10‐month period that included Bennu's perihelion and aphelion. Novel and classical methods were utilized to detect the particles and characterize their orbital and physical properties. Roughly 30% of the observed particle mass escaped to heliocentric orbit. A majority of particles fell back onto the surface of Bennu after ejection, with the longest‐lived particle surviving for 6 days on a temporary orbit. Particle ejection events appear to preferentially take place in the afternoon and evening and from low latitudes, although they can occur at any time or latitude. The reaccumulation of material is biased toward low latitudes resulting in the possible in‐fill of craters and growth of Bennu's equatorial bulge. Of the potential mechanisms behind this activity that were investigated in focused studies, meteoroid impacts, thermal fracturing, and ricochet—but not water ice sublimation—were found to be consistent with observations. While phyllosilicate dehydration was not investigated with a focused study, it remains a possible mechanism. These mechanisms are not unique to Bennu, suggesting that many near‐Earth asteroids may exhibit activity that has gone undetected thus far. Spacecraft missions with wide‐field imagers are encouraged to further characterize this phenomenon.

## Key Points

- Novel and classical methods were applied to detect and characterize particles ejected from near‐Earth asteroid Bennu
- Of the mechanisms investigated, meteoroid impacts, thermal fracturing, and ricochet are consistent with observations
- Some ejected particles escape or temporarily orbit and most reimpact, with implications for Bennu's geophysics and surface properties

## 1 Introduction

On 31 December 2018, the Origins, Spectral Interpretation, Resource Identification, and Security‐Regolith Explorer (OSIRIS‐REx) spacecraft entered orbit around asteroid (101955) Bennu. Only days later, hundreds of millimeter‐ to centimeter‐scale particles were observed emanating from the asteroid's surface (Lauretta et al., 2019). Continued observation of this phenomenon revealed that Bennu is active—that is, experiencing ongoing mass shedding.

Bennu is a near‐Earth asteroid on an orbit that ranges from 0.90 to 1.36 au from the Sun with an eccentricity of 0.20, inclination of 6.0°, and orbital period of 1.20 years. The minimum orbit intersection distance is 0.0033 au, allowing for periodic close approaches between Bennu and Earth. OSIRIS‐REx observations found the visible colors of Bennu to be consistent with a B‐type classification (Clark et al., 2011; DellaGiustina et al., 2019), part of the larger C‐complex whose members are believed to be analogous to carbonaceous meteorites. Surface features on Bennu have albedos between 0.033 and 0.30 with a median of 0.044 ± 0.002 (DellaGiustina et al., 2019; Hergenrother et al., 2019). Bennu is a rubble pile, based on its low measured density of 1,190 kg m^−3^, inferred high bulk porosity, and lack of high surface slopes or substantial topographic relief (Scheeres et al., 2019; Walsh et al., 2019). It is top shaped with a mean diameter of 490 m and sidereal rotation period of 4.296057 ± 0.000002 hr (Barnouin et al., 2019).

Bennu's expected carbonaceous composition, accessible orbit, and existing ground‐based characterization from telescopic data led to its selection as the target of the OSIRIS‐REx mission (Lauretta et al., 2015). A secondary consideration was its B‐type taxonomy, which it shares with other active asteroids such as (3200) Phaethon, 107P/Wilson‐Harrington, 133P/Elst‐Pizarro, 176P/LINEAR, and 249P/LINEAR (Fernández et al., 2017; Hergenrother et al., 2020; Lauretta et al., 2015; Licandro et al., 2011).

Until recent decades, ongoing mass loss was thought to be limited to comets. Historically, the distinction between asteroids and comets was based on their appearance in telescopes. Asteroids appeared as stellar point sources, whereas comets were extended objects because of their tails or comae produced by mass shedding. This observational difference is evident in the origin of their names. The terms “asteroids” and “comets”, respectively, derive from Greek cognates for “starlike” and “hair” or “long‐haired star”. An alternate approach to differentiating between the two populations is based on their orbits. Asteroids mainly have a Tisserand parameter with respect to Jupiter (*T*_J_) > 3, suggesting an origin within the inner solar system, whereas comets predominately have a *T*_J_ < 3, suggesting an origin in the outer solar system (e.g., Levison & Duncan, 1997).

A number of exceptions have challenged such clean distinctions. A class of objects, including the Damocloids, which reside on cometary orbits, have similar colors to active comet nuclei but do not show any detectable activity (Jewitt, 2005, 2015). A temporary or permanent cessation of cometary activity may be due to the loss of a significant fraction of volatile material or perihelia too distant to initiate activity. In the case of (3552) Don Quixote, activity was only detectable at near‐infrared wavelengths (Mommert et al., 2014).

Conversely, more than 20 objects have now been identified on asteroid‐like orbits (*T*_J_ < 3.08; Jewitt et al., 2015) that display mass loss activity. A variety of mechanisms have been proposed to explain these active asteroids (Jewitt, 2012; Jewitt et al., 2015). In some cases, mass loss is the result of discrete near‐instantaneous events due to impacts or rotational instability (Jewitt, 2012; Jewitt et al., 2015). Other bodies show comet‐like volatile sublimation that repeats from orbit to orbit; this activity is dependent on heliocentric distance and is either centered on perihelion or begins near perihelion and ends on the outbound leg of the orbit (Hsieh et al., 2018). Other mechanisms proposed to explain some cases of asteroid mass loss include electrostatic lofting, thermal fracturing, phyllosilicate dehydration, shock dehydration, and radiation pressure sweeping (Jewitt, 2012; Lauretta et al., 2019).

## 2 The OSIRIS‐REx Mission at Bennu

The OSIRIS‐REx spacecraft launched in September 2016 and rendezvoused with Bennu in December 2018 (Lauretta et al., 2017). Bennu was first optically acquired by the OSIRIS‐REx Camera Suite (Rizk et al., 2018) PolyCam instrument on 17 August 2018 from a range of ~2,184,000 km, marking the beginning of the mission's Approach phase. The approach trajectory was designed to optimize initial characterization of the asteroid and its surrounding dynamical environment through a gradual approach to Bennu (Antreasian et al., 2019). Because Bennu was selected as the mission target partly because of its spectral similarity to some active asteroids (Lauretta et al., 2015), the mission plan included observation campaigns for detecting dust and natural satellites.

The first dust searches occurred on 11 and 12 September 2018 at a range of ~10^6^ km, surveying all space within a 35,000‐km radius of Bennu for evidence of unbound dust along the orbit and antisolar vectors. These searches found no evidence of mass loss at a detection upper limit of 150 g s^−1^; high‐density cometary tails or trails would have been visible at that range but not discrete centimeter‐scale particles (Hergenrother et al., 2019). From 23 October to 11 November 2018, a series of 5‐hr natural satellite searches was performed on 10 different dates, as the spacecraft homed in on Bennu at low phase angles. This satellite search yielded null results for objects as small as 24 cm within the Hill Sphere (31 km) and objects as small as 8 cm within 18 km of Bennu (Hergenrother et al., 2019). After OSIRIS‐REx arrived at Bennu on 3 December 2018, the spacecraft conducted a preliminary survey of the asteroid, consisting of 7‐km flybys of the poles and equator. Off‐body detections by the OSIRIS‐REx Laser Altimeter (OLA) (Daly et al., 2017) during this phase suggested that there might be material in the vicinity of the asteroid, but image data, which were not collected simultaneously with the OLA data, did not confirm it.

Particle ejections from the surface of the asteroid were first discovered in images from 6 January 2019, 1 week after the spacecraft entered orbit (Lauretta et al., 2019). This phenomenon was serendipitously captured in NavCam 1 (Bos et al., 2018) images collected for the purpose of optical navigation (OpNav), intended to image background stars while maintaining Bennu in the field of view. The 6 January 2019 ejection event was easily observable in the OpNav images because it involved hundreds of particles (Hergenrother et al., 2020; Lauretta et al., 2019). A later inspection of NavCam 1 images taken before the 6 January 2019 event revealed more subtle evidence of particles in the vicinity of Bennu back to 10 December 2018, though none could be linked directly with the OLA off‐body detections (Chesley et al., 2020).

**Figure 1:**
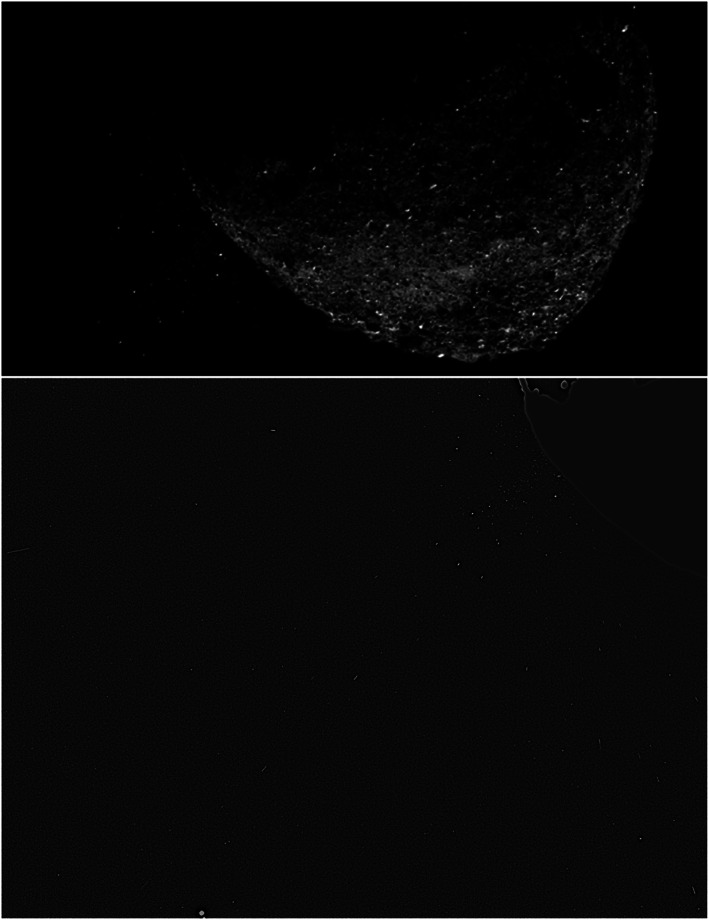
The large particle ejection event seen on 6 January 2019 at 20:56 UTC. (top) In‐field stray light was removed with a variant of unsharp masking (Hergenrother et al., 2020; Samarasinha & Larson, 2014). The saturated disk of Bennu is the smooth feature in the lower right corner. The ejected particles are the majority of the star‐like objects just above the disk of Bennu as well as all trailed objects. The Earth and Moon are the bright double “stars” near the upper left side of the image. (bottom) A hybrid image combining the same 5‐s exposure shown in the top panel to show the particles but without unsharp masking and a short 0.0014‐s exposure to display the nonsaturated surface of Bennu. The two images have start times separated by ~7 s.

**Figure 2:**
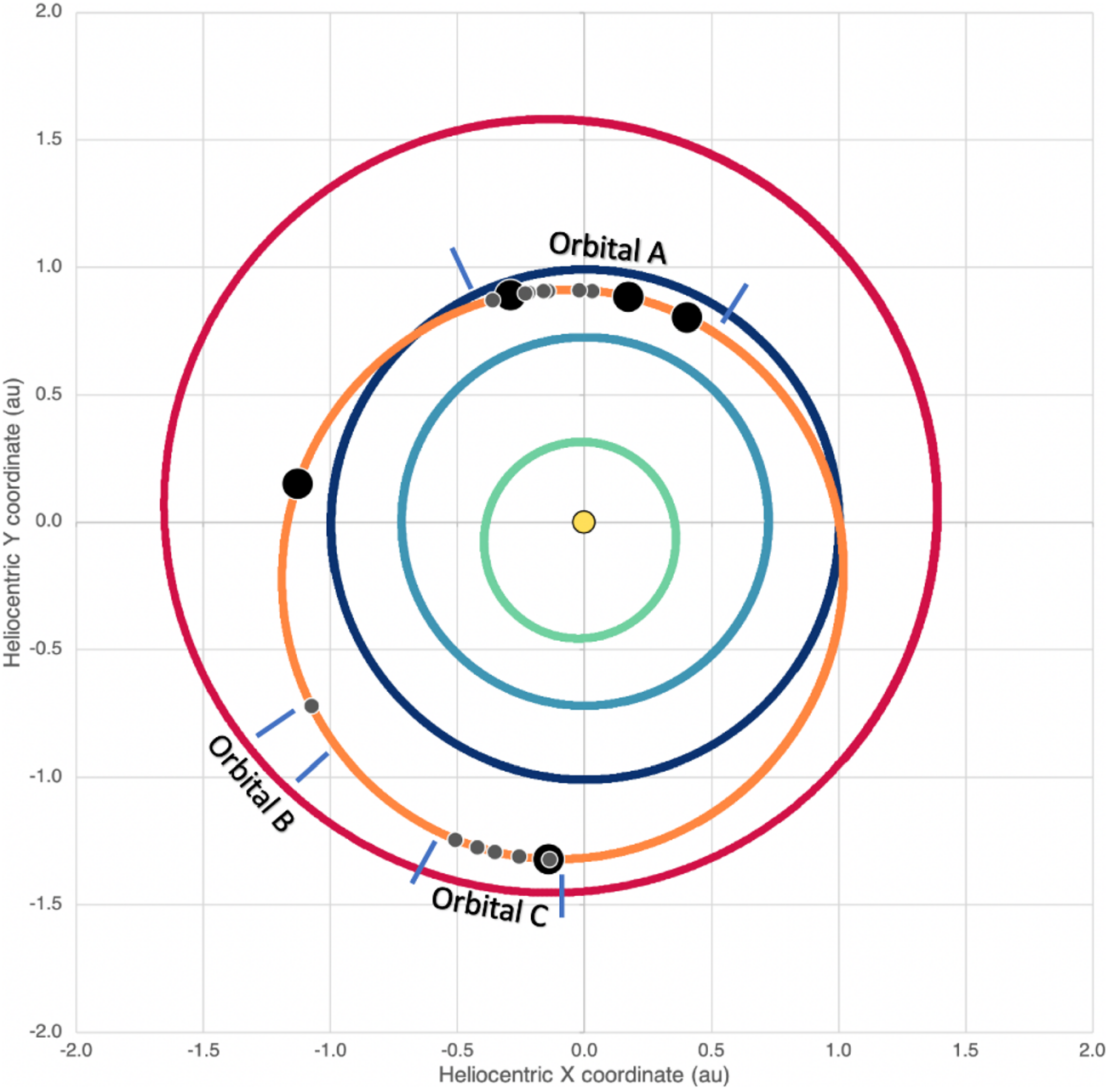
Occurrence of particle ejection events (circles) in the orbit of Bennu (orange). Ejection events of 2 to 19 particles are denoted by small gray circles; events of 20 or more particles are denoted by large black circles. The phases of the OSIRIS‐REx mission that included dedicated particle monitoring (Orbitals A–C) are indicated with blue hatches. The Sun (yellow) and the orbits of Mercury (green), Venus (light blue), Earth (dark blue), and Mars (red) are also shown for reference. Figure is from Hergenrother et al. (2020).

The first image to capture the event on 6 January 2019 showed more than 200 star‐like point‐source and trailed (higher‐velocity) objects located off the limb of Bennu (Figure 1). Objects in common with this first image were observed in a second image taken minutes later, exhibiting apparent motion away from Bennu. This first observation of an ejection event triggered an immediate mission risk assessment, which concluded that the estimated size and velocity of these particles (0.07–3.3 m s^−1^, <1 to 8 ± 3 cm) did not pose risks to the spacecraft in its 1.6 × 2.0 km frozen near‐terminator orbit (Orbital A) (Lauretta et al., 2019). With the decision to remain in orbit, the operations team expedited new observation plans that rolled out an increased imaging cadence in two phases. First, on 10 January 2019, the observation frequency increased from every 2 hr to every 30 min over a 16‐hr window; an observation consists of two 5‐s exposures spaced ~2 min apart. The observation frequency was increased again on 28 January 2019 to every ~20 min over a 16‐hr window (Hergenrother et al., 2020; Lauretta et al., 2019). The high‐cadence particle monitoring completed on 18 February 2019; 10 particle ejection events of varying magnitude were observed during this phase (Figure 2).

After Orbital A, during the mission's Detailed Survey phase from late February to early June 2019, the particle monitoring cadence reverted back to every 2 hr as a ride‐along with the OpNav imaging. The larger ranges and varying phase angles during this phase considerably reduced the NavCam 1 sensitivity to detecting new particle ejection events. However, one large event was observed during this phase on 19 April 2019, at an unusual viewing geometry (Hergenrother et al., 2020). On 12 June 2019, the spacecraft inserted into the closest orbit yet: a near‐circular terminator orbit with a semimajor axis of ~0.9 km (Orbital B). Between 17 and 30 June 2019, the spacecraft executed a dedicated particle monitoring campaign consisting of two overlapping mosaic fields. In addition to observing a constant stream of background particles, one small particle event was detected during this campaign on 18 June 2019 (Hergenrother et al., 2020). An additional longer, dedicated particle monitoring campaign was added to the mission plan: Orbital C, a frozen orbit very similar to Orbital A, with a particle monitoring observation frequency of every 13 min, between 6 August and 16 September 2019. During this campaign, six ejection events were detected (Hergenrother et al., 2020).

As OSIRIS‐REx continues to operate in Bennu's proximity, particle monitoring observations are planned whenever feasible, as long as they do not add any operational risk to the primary mission objective of collecting a sample. A pipeline has been established that automates the detection of potential particles, identifies object pairs between images, reconstructs the event location and timing, and solves for various dynamical and physical properties of each particle.

## 3 Key Findings

### 3.1 Particle Detection, Initial Orbit Determination, and Event Reconstruction

The first step in studying the particles is identifying them among the background stars and the stray light from an overexposed Bennu in the long‐exposure images. The early response to the discovery was a manual identification of particles by registering and blinking or differencing images to see the motion (Hergenrother et al., 2020; Pelgrift et al., 2020) and making use of the fact that, unlike particles, stars in an image pair have the same apparent motion with respect to Bennu. However, an autonomous approach was needed to provide rapid information to the mission on this phenomenon. Liounis et al. (2020) present image processing techniques for autonomous detection of the particles with the Goddard Image Analysis and Navigation Tool, which was in place within weeks of the first recognized particles. This process involves identifying potential objects in an image, filtering the data to reject stars and noise, and assigning a quality code to rate detections on a confidence scale (Liounis et al., 2020). Data such as centroid location, point spread function (PSF) parameters, and signal characteristics are populated into a database for use by the particle analysis team. In scenarios where the automated method misses a detection, particularly for streaked objects and those clustered in the stray light near Bennu, Pelgrift et al. (2020) present methods for extracting the requisite information to supplement the database.

With possible detections extracted for each image, the next step is to identify detections of the same object in multiple frames, which allows for more detailed analysis including event reconstruction (Pelgrift et al., 2020), trajectory fitting (Chesley et al., 2020; Leonard et al., 2020), PSF photometry (Hergenrother et al., 2020), and other scientific investigations. Liounis et al. (2020) provide two solutions for identifying objects traversing multiple frames: (1) by generating visual aids for manual linking and (2) by providing detection data to automated linking algorithms. Additionally, the Catalina Sky Survey moving object detection pipeline (Christensen et al., 2018) has been modified to identify object links between images, as discussed in Hergenrother et al. (2020). These techniques work particularly well for objects captured in multiple images and for tracking the “background” population of longer‐lived objects.

Once detections are linked across multiple frames and populated into the database, the dynamics of recently ejected and longer‐lived particles can be estimated based on statistical orbit determination techniques, presented by Leonard et al. (2020) and Chesley et al. (2020).

However, for several of the observed events, the only detections of ejected particles come from the first two images taken immediately after the event. Without three or more observations of each particle, traditional orbit determination is not possible, and thus, a new method was developed to estimate particle positions, velocities, and time and location of origin when only sparse data are available (Pelgrift et al., 2020). For ejection events that do have three or more detections per particle, Leonard et al. (2020) present initial orbit determination techniques to rapidly estimate particle trajectories, excess velocity necessary to induce the particle ejection from the surface, and uncertainty quantification of ejection locations.

### 3.2 Detailed Characterization of Particle Behavior and Properties

**Figure 3:**
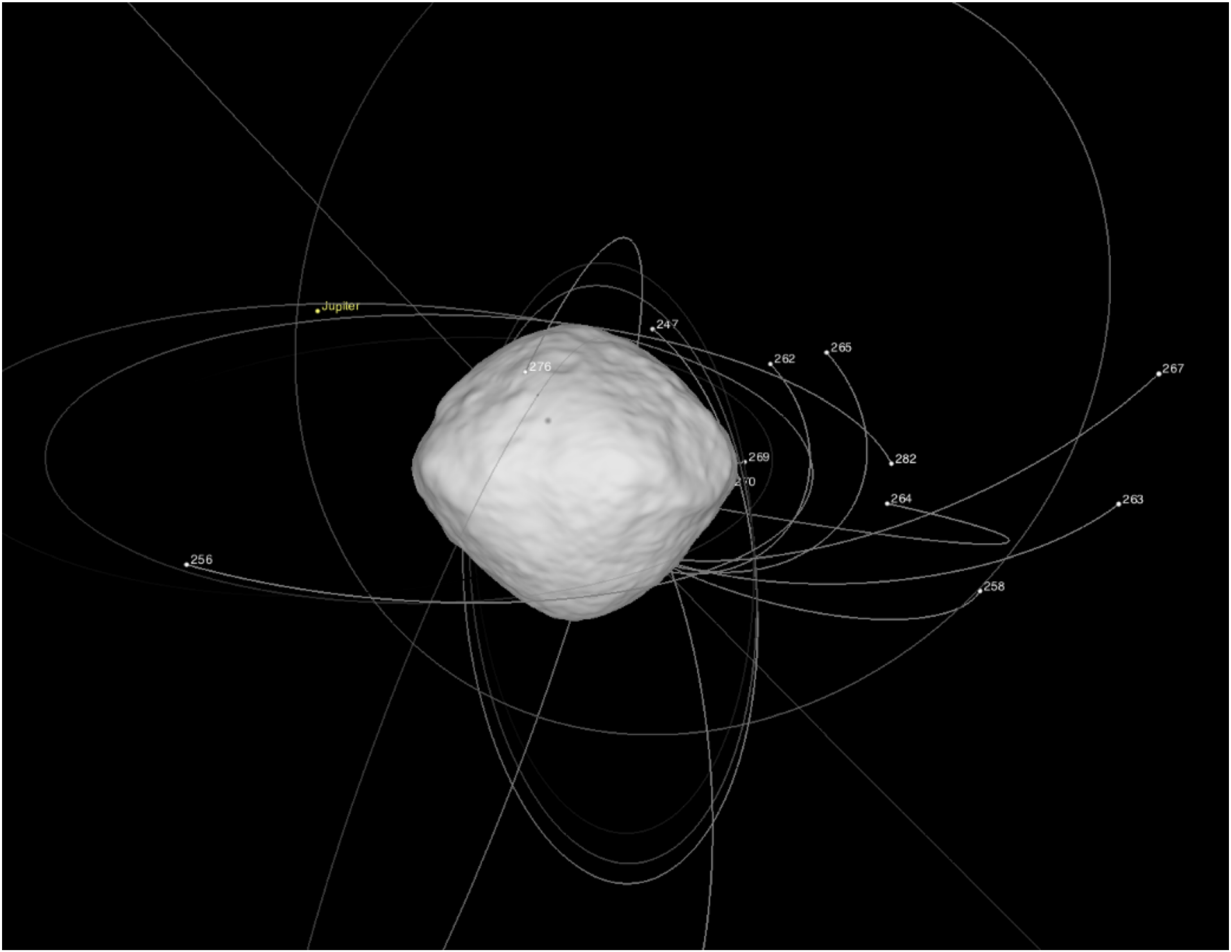
The near‐environment of Bennu shown shortly after the ejection event of 28 August 2019. Particle positions are shown as white dots and labeled with identifiers from Chesley et al. (2020) and Hergenrother et al. (2020). Particle trajectories extend back 18 hr or until the moment of ejection, which ever time is shorter.

**Figure 4:**
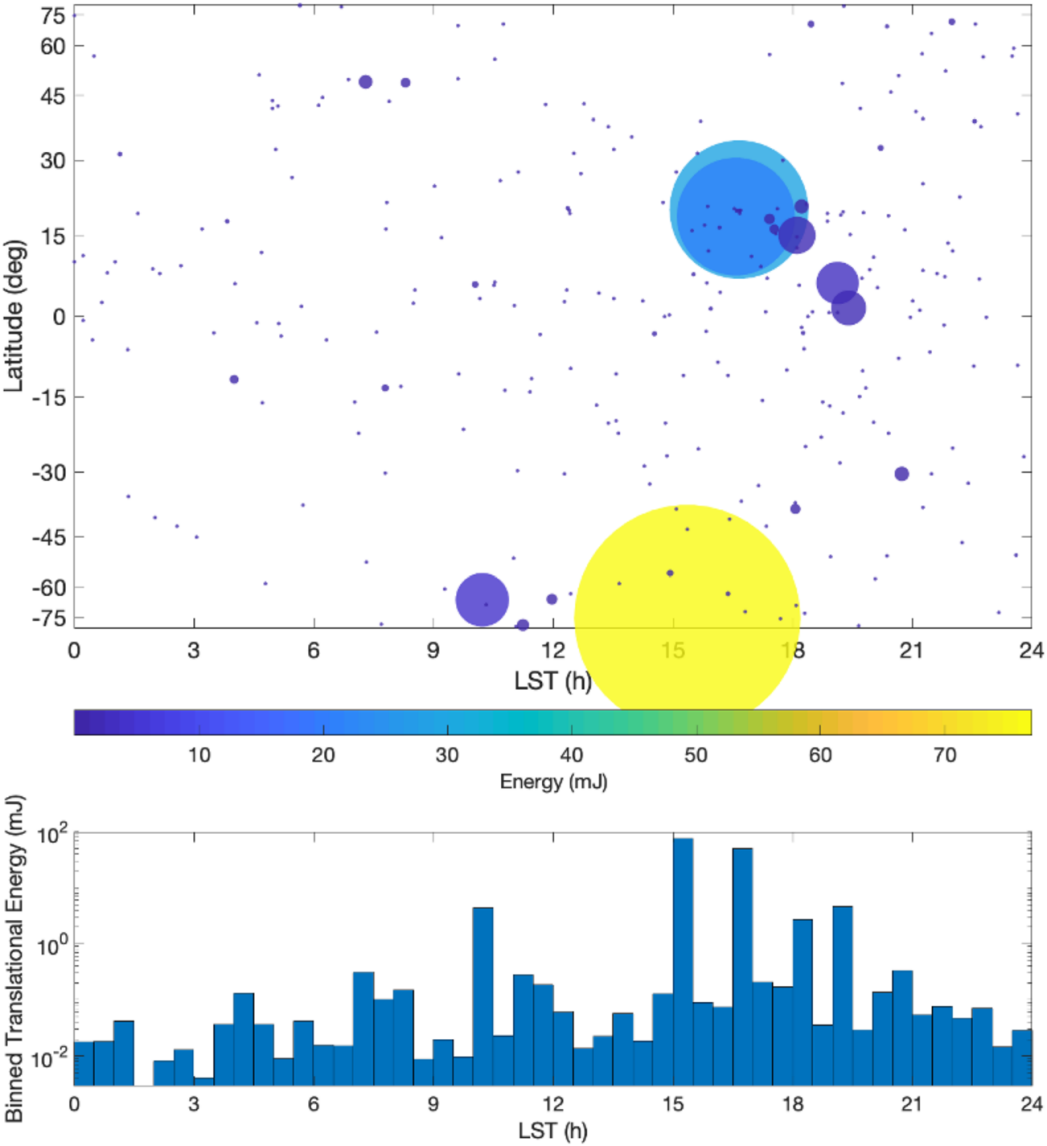
Observed translational energy versus local solar time. Local solar time is shown in hours with 0/24 hr corresponding to local midnight and 12 hr to local noon. (top) Local solar time versus Bennu latitude. The area of the circles corresponds to the observed translational energy. (bottom) Observed translational energy versus local solar time in half‐hour bins.

Chesley et al. (2020) linked multiple particle tracks to extend the arcs of many objects, allowing for long‐arc estimation of particle trajectories and dynamics, for a data set spanning December 2018 to September 2019 (including Bennu perihelion and aphelion) (Figure 3). They report trajectories for 313 particles. This study enabled the estimation of Bennu's gravity field spherical harmonic coefficients through Degree 8 and resolved nonuniform mass distribution through Degree 3. Furthermore, from the estimated perturbations on the particles due to solar radiation pressure, effective area‐to‐mass ratios were calculated. By assuming that particles are rotating oblate ellipsoids and incorporating photometric measurements from Hergenrother et al. (2020), Chesley et al. (2020) find a median axis ratio of 0.27 indicative of flake‐like shapes and diameters for equivalent‐volume spheres ranging from 0.22 to 6.1 cm, with a median of 0.74 cm. They further find that most particle ejection events take place at local solar times in the afternoon and evening (12:00 to 24:00)—similar to the findings of Pelgrift et al. (2020), Leonard et al. (2020), and Lauretta et al. (2019)—but that particles not associated with observed ejection events can have origin points that occur at any local solar time (Figure 4). Of the observed particles included in the Chesley et al. (2020) analysis, 65% followed suborbital trajectories, 20% underwent more than one orbital revolution, and 15% directly escaped on hyperbolic trajectories. Hergenrother et al. (2020) expanded the Chesley et al. (2020) data set to include observed particles from the large ejection events (Leonard et al., 2020; Pelgrift et al., 2020) and found that ~30% of the observed particle mass escaped into heliocentric orbit. The longest‐lived observed orbiting particle had a lifetime of ~6 days.

Complementarily, McMahon et al. (2020) modeled the orbital dynamics of simulated particles launched under the conditions of the largest observed ejection events (6 January, 19 January, and 11 February 2019), finding that many simulated particles on hyperbolic orbits do not escape immediately. Particles launched toward the Sun are acted upon by solar radiation pressure, which pushes them back toward and past Bennu before they eventually escape into heliocentric orbit. Due to solar radiation pressure, some orbital particles on very high trajectories (semimajor axis ~20 km) enter orbit. The simulated population after an event quickly decays, with nearly half of the particles reimpacting the surface of Bennu within the first day. By 10 days, most of the population in the simulation has either reimpacted or escaped. A small subset of the simulated population survives for much longer, with 0.06% of particles remaining in orbit after one Bennu year. The lack of OSIRIS‐REx detection of particles with lifetimes of weeks to months may be a result of observational bias. These particles are on very high trajectories (semimajor axis ~20 km) (McMahon et al., 2020) and spend a large fraction of their orbit too far from the spacecraft to be detected or outside the NavCam 1 field‐of‐view.

Hergenrother et al. (2020) used PSF photometry to determine the physical properties of the particles and ejection events observed between December 2018 and September 2019, the same time period as in Chesley et al. (2020), encompassing Bennu's perihelion and aphelion. The approach of this study relies on a few inputs from other work in the special collection, such as estimated range to the particles and estimated area‐to‐mass ratios (Chesley et al., 2020; Leonard et al., 2020; Pelgrift et al., 2020). The technique involves first measuring phase functions for a subset of particles with 40 or more photometric measurements spanning a phase angle range of >20° and then using the derived phase functions to determine the absolute magnitude, or intrinsic brightness, of the observed particles. Absolute magnitudes can be converted to sizes and then to masses via the estimated area‐to‐mass ratios from Chesley et al. (2020). Finally, masses and ejection velocities are converted to translational energies, and characteristics of mass loss can be estimated. Only a single particle showed possible evidence of a resolved rotational lightcurve. Nearly all particles show no evidence of rotation suggesting rotation periods of much less than the 5‐sexposure time (Hergenrother et al., 2020). Rotational kinetic energy may be orders of magnitude greater than the observed translational kinetic energy. The phase function, with a phase coefficient of 0.013 ± 0.005 mag deg^−1^ for high‐quality particles, are unlike those seen for ensemble asteroid surfaces and more similar to laboratory measurements of individual centimeter‐sized terrestrial samples. This study finds a possible increase in activity close to perihelion, but ejection events occur close to aphelion as well, and observation bias remains a key limitation to be addressed in future work (Figure 2). A decrease of ~70% in daily ejection mass was observed at aphelion compared to perihelion. The daily translational energy rate was approximately unchanged between perihelion and aphelion.

### 3.3 Mechanisms of Ejection

A number of particle ejection mechanisms were preliminarily investigated by Lauretta et al. (2019), including rotational disruption, electrostatic lofting, ice sublimation, phyllosilicate dehydration, thermal stress fracturing, meteoroid impacts, and secondary surface impacts by reimpacting particles (ricochet). Phyllosilicate dehydration, thermal stress fracturing, and meteoroid impacts were found to be the most plausible mechanisms for the largest observed events, with secondary impacts possible for smaller events. The special collection dives further into several of these possible mechanisms.

High‐resolution modeling of the global surface and subsurface temperatures of Bennu to evaluate the plausibility of water ice sublimation and thermal fracturing as ejection mechanisms was performed by Rozitis et al. (2020). Only ~1,856 m^2^ or ~0.2% of Bennu's surface was found to have orbit‐averaged subsurface temperatures that are sufficiently cold for water ice to remain stable over geological timescales, if buried within the top few meters of the surface. All of these locations are in Bennu's polar regions at latitudes above ~60° North and South. If Bennu's orbit and pole orientation remained constant, such ice could remain stable for up to a billion years. Centimeter‐scale polar cold traps could support stable millimeter‐thick layers of surface water ice on ~10^3^‐year timescales. If water ice sublimation were the primary mechanism of particle activity on Bennu, ejection sites would preferentially occur at high latitudes. However, particle ejection has also been observed to originate from the much warmer equatorial region of Bennu (Chesley et al., 2020; Lauretta et al., 2019; Pelgrift et al., 2020) (Figure 4). Thus, water ice sublimation is not the primary process ejecting particles from the surface of Bennu.

However, evidence of exfoliation—or the flaking and disaggregation of thin layers of surface material, often associated with thermal stress—has been identified on Bennu's boulders (Lauretta et al., 2019; Molaro, Walsh, et al., 2020). Molaro, Hergenrother, et al. (2020) simulated thermal fracturing for “dense” and “porous” boulders with densities of 2,510 and 1,812 kg m^−3^, respectively. Thermally induced stress in both boulder types is larger than the tensile strength of boulders on asteroid (162173) Ryugu (Grott et al., 2019), the C‐type near‐Earth asteroid target of the Hayabusa2 mission. Models of the exfoliation of boulder surface layers near perihelion produce tens of centimeter‐scale particles from the smallest boulders to thousands in the largest boulders. Such particle numbers are comparable to those observed across all observed ejection event scales (one to hundreds of centimeter‐scale particles). Modeled particle ejection velocities range from ~0.3 to 0.8 m s^−1^ for dense boulders and up to maximum of ~2 m s^−1^ for porous boulders, which is roughly consistent with observed particle velocities (0.05 to 3.3 m s^−1^). The thermal strain energy at a boulder's surface peaks in the afternoon and is an order magnitude higher than at other times of day. This matches the observed tendency toward ejection events in the local afternoon (Chesley et al., 2020; Lauretta et al., 2019; Leonard et al., 2020; Pelgrift et al., 2020) (Figure 4).

Supporting the findings of Molaro, Hergenrother, et al. (2020), Rozitis et al. (2020) found large diurnal temperature variation and likelihood of rock thermal fracture at all latitudes on Bennu owing to the extreme ruggedness of its shape. If thermal fracturing is the primary mechanism behind particle ejection, it could occur from any latitude on Bennu's surface, consistent with observations (Chesley et al., 2020; Lauretta et al., 2019; Leonard et al., 2020; Pelgrift et al., 2020). However, as the amount of surface area that reaches maximum diurnal temperature variation is greatest near the equator, particle ejection is more likely at low latitudes in the afternoon (Chesley et al., 2020; Rozitis et al., 2020).

The strain energy available in a surface boulder peaks at perihelion, when diurnal temperature variations are greatest, and decreases as Bennu moves toward aphelion (Molaro, Hergenrother, et al., 2020). The annual exfoliation stress peaks roughly 30 days prior to perihelion (Molaro, Hergenrother, et al., 2020). If thermal fracturing was the primary ejection mechanism, the combination of these two factors would cause large, high‐energy events to preferentially occur at or approaching perihelion. Less frequent, lower‐energy events would be possible at larger heliocentric distances, including at aphelion.

**Figure 5:**
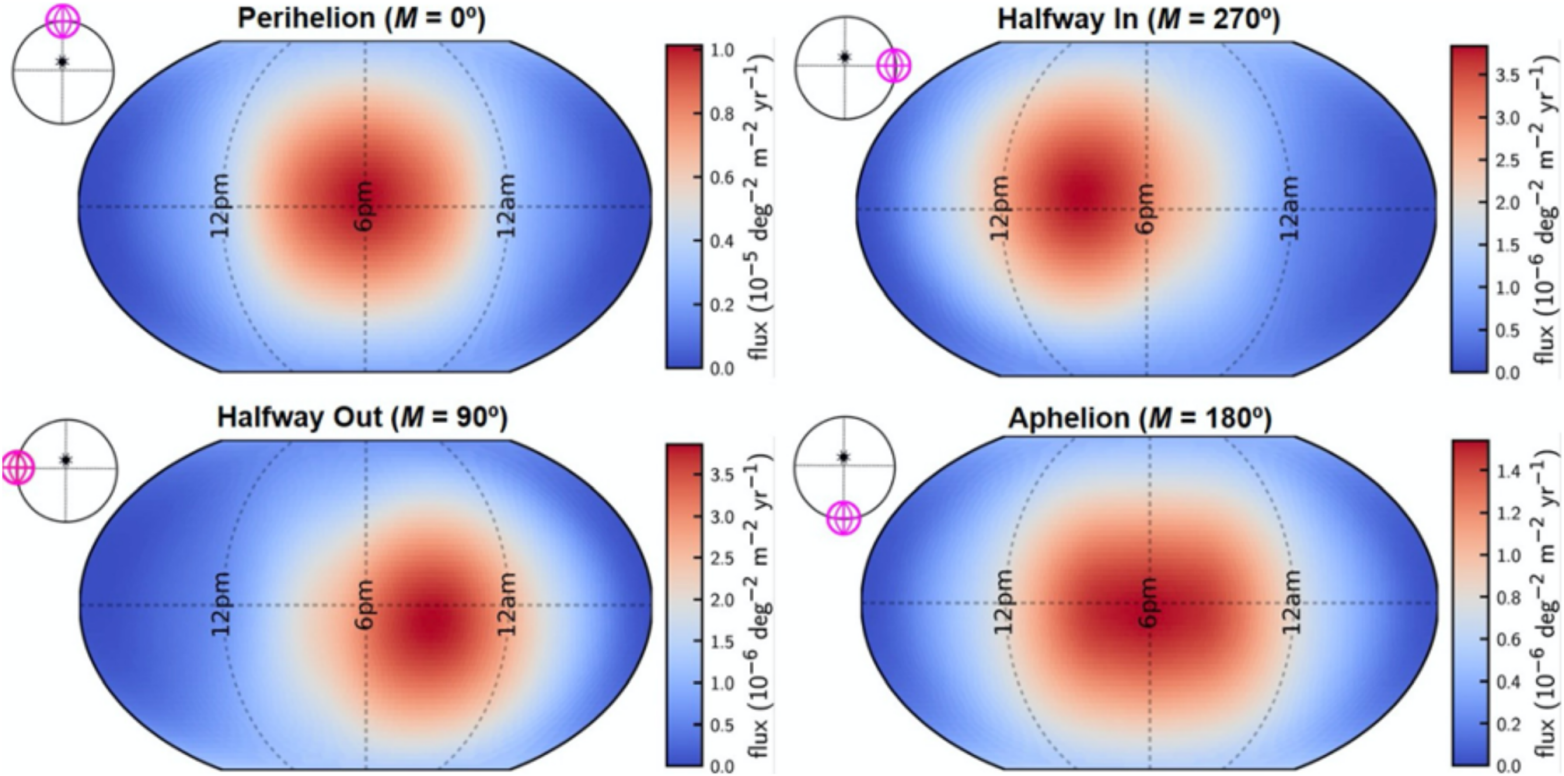
The meteoroid impact flux across the surface of Bennu at different points in its orbit. The scale bars decrease by an order of magnitude between the plot at perihelion and those further from the Sun. Bottke et al. (2020) found that the flux is greatest at perihelion. Impacts skew to the evening on the outbound part of the orbit and to the early afternoon on the inbound part. A preference for ejection from the afternoon and evening side of Bennu matches the findings of Chesley et al. (2020) shown in Figure 4. This figure is from Bottke et al. (2020).

Hypervelocity impacts were previously identified as the cause of discrete mass loss events on some active asteroids, such as (596) Scheila and P/2016 G1 (PANSTARRS) (Moreno et al., 2016). In those cases, the estimated size of the impactor was tens of meters. Bottke et al. (2020) determined that much smaller meteoroid material is capable of striking Bennu and producing the observed particle events. This study employed the NASA Meteoroid Engineering Model to simulate the cometary sporadic meteoroid population (McNamara et al., 2005; Moorhead et al., 2019). The simulations indicate that near perihelion, Bennu is impacted once every 2 weeks by a meteoroid with an average impact speed of 43 km s^−1^ and 7,000 J of average impact kinetic energy. The impact points on the surface of Bennu are biased toward the late afternoon, corresponding to the leading edge of Bennu in its orbital motion (Figure 5). As with thermal fracturing, this is consistent with the local solar time distribution of particle ejection events (Chesley et al., 2020) (Figure 4). Cometary meteoroids can eject sufficient mass to explain the sizes of the largest observed particles if the rocks and boulders on Bennu are structurally weak and highly porous. Bottke et al. (2020) predict that fewer energetic particle ejection events occur as Bennu moves toward aphelion and that the timing of the events skews toward the evening and morning on the outbound and inbound side of its orbit, respectively.

About two thirds of the particles analyzed by Chesley et al. (2020) returned to impact Bennu's surface. Lauretta et al. (2019) proposed that such secondary impacts could result in the particle bouncing or even the lofting of some number of particles that are dislodged by the secondary impact. Indeed, Chesley et al. (2020) identified one case of a particle bouncing from the surface, and so the phenomenon has been confirmed, and yet the fact that few bounces have been recognized suggests that they are not likely to be a major source of ejected particles. Moreover, barring a self‐sustaining chain of secondary impacts, there must be at least one additional mechanism to initiate the reimpact sequence.

**Figure 6:**
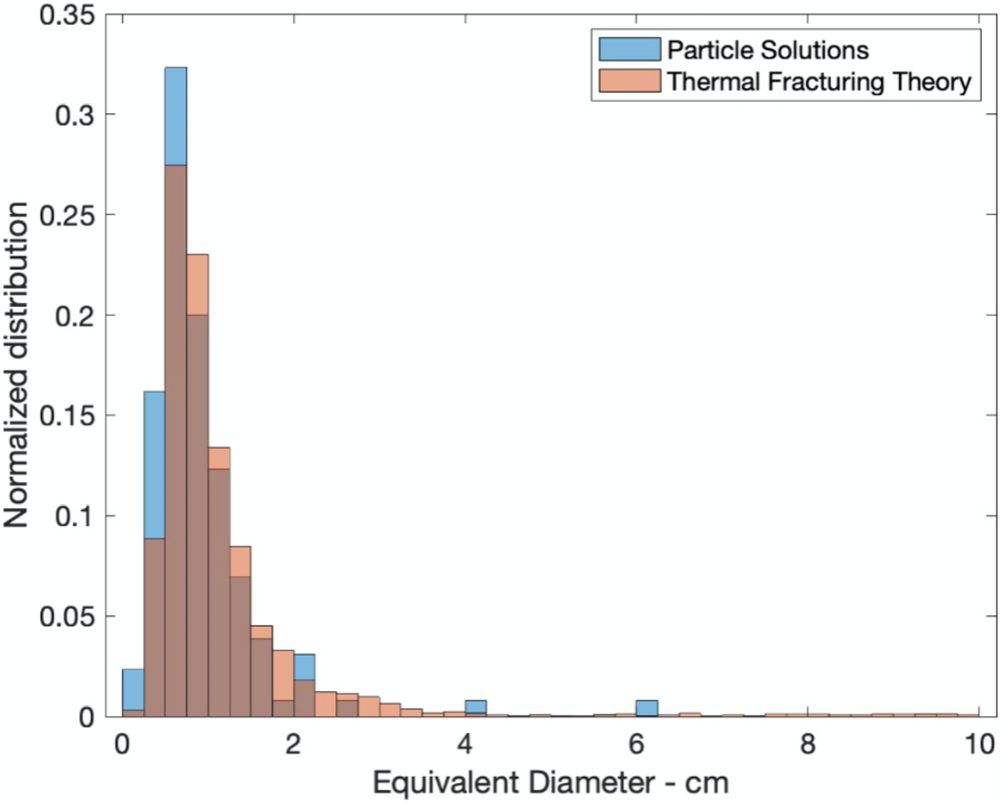
Comparison of the theoretical and observed distribution of particle volume‐equivalent spherical diameters. The theoretical size distribution based on the thermal fracturing model of Molaro, Hergenrother, et al. (2020) is depicted in red. The observed distribution is by Chesley et al. (2020). Figure is from Chesley et al. (2020).

Predicted particle size distributions from ejection events produced by thermal fracturing (Molaro, Hergenrother, et al., 2020) closely match the observed size distribution found by Chesley et al. (2020) (Figure 6). The preference for ejections in the afternoon and evening also matches that expected for thermal fracturing. However, the predictions for events produced by meteoroid impacts are also consistent with findings of Chesley et al. (2020): Bottke et al. (2020) predict most ejections will be in the afternoon and evening, with an enhancement in the equatorial regions. Multiple mechanisms may be responsible for the particle ejection phenomenon. For example, thermal fracturing may be liberating particles that are then lofted by meteoroid bombardment (Chesley et al., 2020).

Phyllosilicate dehydration was not the topic of a focused article within this special issue but was addressed in Lauretta et al. (2019) for Bennu and in Jewitt (2012) for other active asteroids. Water‐bearing minerals have been identified within some carbonaceous meteorites including the CM‐types which are the closest spectral match to Bennu (Clark et al., 2011; Hamilton et al., 2019). Evolved gas analysis experiments on the CM meteorite Murchison found that volatile release can occur at the temperatures slightly above those reached on Bennu's surface (Gibson, 1974; Gibson & Johnson, 1972; ten Kate et al., 2010). Thermally induced mechanical stresses may generate adsorbed water. The concentration of adsorbed water within cracks and pores in boulders could enhance the effects thermal fracturing (Molaro, Hergenrother, et al., 2020) or possibly could result in outbursts or particle lofting due to gas drag (Jewitt & Li, 2010). The OSIRIS‐REx Visible and Near‐InfraRed Spectrometer spectra showed a spectroscopic signature of the presence of water‐bearing minerals on the surface of Bennu (Hamilton et al., 2019) bolstering the possibility of dehydration as a driver of particle activity on Bennu.

Electrostatic lofting occurs when the electrostatic force is larger than the gravity and cohesion that bind particles to the surface. Submillimeter particles can be electrostatically lofted to a height of meters from the surface of small asteroids (Hartzell, 2019; Hartzell & Scheeres, 2013). Particles detached from the surface may then be swept away by solar radiation pressure. Hartzell et al. (2019) found that particles lofted from a Bennu‐like asteroid should have smaller sizes and velocities than those of the observed ejection events. Electrostatic lofting is not a plausible mechanism for the largest observed events on Bennu (Lauretta et al., 2019), but analysis of how this phenomenon may be operating on Bennu is ongoing.

### 3.4 Geophysical Implications of Particle Ejection

The ongoing ejection of particles could have a long‐term effect on the rotation, orbit, and regolith distribution of Bennu. Scheeres et al. (2020) analyzed the possible consequences relative to the Yarkovsky and Yarkovsky‐O'Keefe‐Radzievskii‐Paddack (YORP) effects. The Yarkovsky effect is a nongravitational force acting on a rotating body that causes a change in semimajor axis (Chesley et al., 2014). YORP is responsible for changes in the rotation state of an asteroid (acceleration or deceleration) due to scattering of solar radiation and thermal emission (Rubincam, 2000).

In their model, Scheeres et al. (2020) assumed a scenario involving 10‐cm‐diameter particles (at the high end of the range given in Lauretta et al., 2019) with a density of 2,000 kg m^−3^ escaping at 1 m s^−1^ in the transverse direction along the orbit of Bennu. The resulting change in velocity is smaller by a factor of 3,000 to 7,000 than that produced by the daily Yarkovsky effect. The high fractional precision of the Yarkovsky effect on Bennu may require the consideration of particle ejection for its modeling. If particles are assumed to be uniformly removed from the asteroid surface and deposited onto the equator, the rate of ejected mass required to be ejected to reach the magnitude of the YORP effect is equivalent to eight 1‐cm particles per second. Such a rate is much larger than that observed at Bennu. For the observed ejections, the maximum rate may be equivalent to only 0.04% of the measured YORP effect. The direction of any particle‐induced change in Bennu's rotation rate may be used to differentiate between production mechanisms. If ejections are due to thermal fracturing, which would preferentially occur on western‐facing boulders, Bennu would tend to experience a spin‐up in rotation rate. Conversely, if meteoroid impacts are the primary mechanism, particles ejected to the east would preferentially escape relative to particles ejected to the west, tending to decrease Bennu's rotation rate and masking the strength of the overall YORP effect. However, such directionality is not evident from the current data set.

The launch and removal of particles from the surface of Bennu affect both the distribution of regolith and the shape of Bennu. McMahon et al. (2020) found that the removal of small (diameter ~ millimeters) particles by ejection could lead to a deficit of this size range on Bennu's surface. Cumulative size frequency distributions of the largest ejection events show an apparent deficit in particles smaller than 0.7 cm, though observational biases may also explain the result (Hergenrother et al., 2020). However, global mapping of Bennu provides little evidence for latitudinal size sorting of particles (Walsh et al., 2019), suggesting that other processes may have driven or dominated the depletion of fine material at low latitudes.

Reimpacting particles preferentially settle at low latitudes, which could in‐fill craters and enlarge Bennu's equatorial bulge without requiring landslides (Chesley et al., 2020; McMahon et al., 2020). This preference for reaccumulation at low latitudes was also found by Winter & Amarante (2017) for Bennu and Winter et al. (2020) for (153591) 2001 SN263, a B‐type near‐Earth asteroid with a similar shape to Bennu's.Particle reimpact may be causing a redistribution of mass from polar and middle latitudes to lower latitudes, resulting in a low‐latitude accumulation of small particles (<5 cm) (Scheeres et al., 2020; Winter & Amarante, 2017). Scheeres et al. (2019) identified a pronounced reduction in surface slope within Bennu's rotational Roche lobe in the equatorial region, which could be explained by the transfer of ejected material to lower latitudes. For the measured particle ejection rate of 10^4^ to 10^5^ particles per year (Hergenrother et al., 2020), the top ~0.2 to 2 m of the surface would be processed in 10 ×10^6^ years. Infalling particles ricocheting, or bouncing, off the surface experience a moderation in their eccentricity and inclination, also resulting in preferentially landing at low latitudes (Chesley et al., 2020).

## 4 Bennu's Activity and Its Place Within the Population of Active Asteroids

A possible relationship between Bennu and active asteroids was recognized prior to the observation of particle ejection (Hergenrother et al., 2020; Lauretta et al., 2015). The mass loss observed at Bennu may represent the low end on the continuum of active objects. Hergenrother et al. (2020) estimated the mass loss rate of Bennu averaged over one Bennu orbit (1.2 years) at ~10^−4^ g s^−1^, orders of magnitude smaller than measured for other active objects. Peak dust production rates for highly active objects such as comet C/1995 O1 (Hale‐Bopp) have been measured as large as 10^9^ g s^−1^ (Jewitt & Matthews, 1999), while the very‐low‐activity comet 209P/LINEAR produced only 10^3^ g s^−1^ (Schleicher & Knight, 2016; Ye et al., 2016). Mass loss rates for active asteroids experiencing volatile sublimation range from 10^1^ to 10^3^ g s^−1^ (Jewitt et al., 2015).

Active asteroid (3200) Phaethon has a B‐type taxonomy and mass loss rate of 10^2^ to 10^3^ g s^−1^ near perihelion (Hui & Li, 2016); however, it is larger (6.2‐ vs. 0.49‐km diameter) and travels closer to the Sun (0.140 vs. 0.895 au) than Bennu. By assuming that the ejection process is roughly proportional in strength to heliocentric distance (*r* ^−2^) and surface area, Hergenrother et al. (2020) normalized Benn's perihelion distance and surface area to that of Phaethon and found that Bennu's normalized mass loss rate is ~1 g s^−1^. If the ejection process increased at *r* ^−3^ or *r* ^−4^, as is typical for comets (Green et al., 2001), then Bennu's activity, when mapped to 0.14 au, would rival that of Phaethon. It is unlikely that activity at Phaethon is driven by the sublimation of near‐surface ices; rather, the leading mechanisms for particle production are thermal stress fracturing and radiation pressure sweeping (Jewitt & Li, 2010). The nondetection of hydrated minerals on the surface of Phaethon also casts doubt on phyllosilicate dehydration as an activity mechanism (Takir et al., 2020). A second small‐perihelion object has been found to experience activity. Though classified as a comet, 322P/SOHO has a perihelion distance of 0.053 au and is an S‐ or V‐type asteroid (Knight et al., 2016), confirming that mechanisms such as thermal stress fracturing and radiation pressure sweeping may be active on noncarbonaceous objects. Even so, Granvik et al. (2016) found that it is predominantly the small, dark asteroids that disrupt when reaching low perihelion distances, suggesting that primitive, volatile‐rich asteroids are more vulnerable to disintegration, and presumably mass loss, at a given heliocentric distance.

A common trait of small bodies experiencing mass loss is the production of meteoroid streams and their resulting meteor showers. While no meteors from Bennu have yet been identified, Bennu's ejected particles are currently able to reach Earth (Kováčová et al., 2020; Ye, 2019). A number of carbonaceous near‐Earth asteroids have been associated with meteor showers observable on Earth. Two of the strongest annual showers, the Geminids and Quadrantids, originate from inactive or low‐activity carbonaceous asteroids. The aforementioned Phaethon is the parent of the Geminid shower. (155140) 2005 UD, which is dynamically related to Phaethon, is the parent of the Daytime Sextanids (Jewitt & Hsieh, 2006). The Quadrantid shower is produced by (196256) 2003 EH_1_, which is currently an inactive C‐type asteroid but was observed as a bright comet in 1490 (Jenniskens, 2004; Kasuga & Jewitt, 2015). The B‐type asteroid 2001 YB_5_ is responsible for the minor Southern *δ* Cancrids shower (Dumitru et al., 2017; Meng et al., 2004). Close to 50 other asteroids, including Q‐, S‐, V‐, and X‐types, have been identified as meteoroid producers, demonstrating that meteoroid production via mass loss is not confined to carbonaceous asteroids (Dumitru et al., 2017). The size frequency distribution of large particle ejection events on Bennu is similar to that of the Geminid and Quadrantid meteor showers (Blaauw et al., 2011; Hergenrother et al., 2020). Activity like that seen at Bennu may be a significant contributor to meteoroid production. The detection of meteor showers associated with other near‐Earth asteroids would act as an indirect measure of Bennu‐like activity.

## 5 Future Directions for Asteroid Activity Research

Direct observations of millimeter‐ to centimeter‐scale particles in interplanetary space are scarce. The extensive data set acquired by OSIRIS‐REx while in record‐setting proximity to asteroid Bennu has provided knowledge about active asteroids that could not have been obtained from Earth (Hergenrother et al., 2020). This data set begins to fill the gap between direct measurement of micron‐scale particles and meter‐scale and larger particles observed above Earth's atmosphere. The largest ejection events are many orders of magnitude smaller than required to be detected by Earth‐based or near‐Earth space‐based telescopes, and as such the future study of this phenomenon requires observations by spacecraft. Although the OSIRIS‐REx data set provides valuable evidence, biases may have been introduced by the variation in detection methods and observing circumstances over the course of the observations.

Interest in deploying spacecraft to active asteroids was high before the discovery of activity at Bennu. A number of missions to active asteroids with volatile‐driven activity have been proposed, though none have been selected for flight (Jones et al., 2018; Meech et al., 2015; Snodgrass et al., 2018; Zhang et al., 2019). Phaethon is the target of the upcoming JAXA DESTINY+ mission, which includes a dust analyzer among its suite of instruments (Krüger et al., 2019). Predictions of the distribution of ejected material around Phaethon due to meteoroid impacts have been made by Szalay et al. (2019).

Many of the proposed mechanisms, such as thermal fracturing and meteoroid impacts, are not limited to carbonaceous objects and open the possibility that some level of Bennu‐like particle activity is occurring on all near‐Earth asteroids. While the search for mass loss activity and satellites is a common exercise for all small body missions, only OSIRIS‐REx was sensitive to particles with diameters less than 10 cm (Fuse et al., 2008; Hergenrother et al., 2020; McFadden et al., 2015, 2018; Watanabe et al., 2019). The null detection of particles at other asteroids does not preclude the existence of similar unresolved activity at those bodies. Future asteroid missions would benefit from the use of wide‐field imagers to monitor the environment around their targets to detect millimeter‐ to centimeter‐scale particles.

## Data Availability

The data presented come from the papers in this special collection and from online sources (https://figshare.com/s/19e444f5f6fc9793c919 and https://figshare.com/s/08d550cfd745de1a9a60). The image in Figure 1 was produced by processing image 20190106T205618S792_ncm_L0.fits, available via the Planetary Data System in the TAGCAMS section of the OSIRIS‐REx bundle (https://sbn.psi.edu/pds/resource/orex/tagcams.html).
